# Leadless pacing in young patients

**DOI:** 10.1093/eurheartjsupp/suae090

**Published:** 2025-03-24

**Authors:** Paul Richard Roberts, Saverio Iacopino

**Affiliations:** Faculty of Medicine, University of Southampton, University Road, Southampton SO17 1BJ, UK; GVM Care & Research, Maria Cecilia Hospital, Cotignola, RA, Italy

**Keywords:** Leadless pacemaker, Young adult, Paediatrics, Adult congenital heart disease, Vasovagal syncope, Complications

## Abstract

Leadless pacing has been established as an effective and safe therapy. The majority of published data relates to an older population. However, this therapy may be particularly attractive for a younger (<40 years) population where the complications of transvenous devices can be enhanced. These include having leads in the vasculature for a longer period of time and the need for more generator changes over a longer time frame that may increase the rate of complications, e.g. infection. There is increasing evidence that leadless pacing is safe and effective in a younger population that may include specific patient groups such as those that require infrequent pacing, e.g. cardioinhibitory vasovagal syncope. Additionally, leadless pacing may be particularly suited to patients with adult congenital heart disease where conventional access with transvenous devices may be challenging. There are also some other groups such as neuromuscular disorders associated with conduction system disease that may benefit. The paediatric population is a further group that offers many challenges for pacing and so may be considered for leadless pacing. There is an increasing evidence base for the use of a superior jugular approach in this population rather than the conventional femoral approach. Further evidence to support leadless pacing would be enhanced with prospective randomized controlled studies in the population under the age of 40 years.

## Introduction

Leadless pacemakers (LP) are now a recognized alternative to transvenous pacemaker (TP) systems. There are several perceived benefits of LP that include avoidance of long-term leads in the vasculature and across the tricuspid valve, reduced generator changes, absence of a visible device on the chest wall, and low infection rates. It is arguable that all these benefits might be considered particularly attractive in a young population requiring pacing for more years than an older group. However, in the young, the issue of how LP devices are managed at the end of battery life is an important factor to consider with this therapy.

## Evidence base for leadless pacing in the young

The first published data on LP were the experience of the Nanostim device (St Jude Medical, CA, USA) that included patients aged between 19 and 96 years with a mean of 75.8 ± 12.1 years.^1^ These data were followed shortly after with the Micra (Medtronic, MN, USA) investigational device exemption (IDE) study that reported a similar age range of 19–94 years with a mean of 75.9 ± 10.9 years.^2^ In the Post Approval Registry (PAR) study of the Micra device, the demographics of the population were on average older (75.6 ± 13.5 years) than a control group of TP pacemakers (71.1 ± 12.1 years, *P* < 0.001).^3^ The age range in the PAR was 13–101 years. The Aveir device (Abbott Medical, CA, USA), which has both a ventricular and atrial component, reported a mean age of 69.2 ± 13.5 years in its pivotal study though the age range was not reported.^4^

Data from the Micra IDE study, PAR study, and Continued Access study included 2737 patients implanted with the device and reported age-stratified peri-procedural outcomes and intermediate-term follow-up.^5^ One hundred and twenty-four patients had the Micra device implanted under the age of 50 years. In this age cohort, there were less comorbidities including lower incidence of atrial fibrillation (AF), congestive heart failure, chronic obstructive pulmonary disease, hypertension, and diabetes when compared with older patients. In the young cohort, the primary indication for pacing was syncope compared with bradycardia with AF in the older population. Median pacing percentage was lowest in the <50 group at 0.6% compared with the oldest group (>80 years) where it was 68.5%. There was no difference in major complication rates between the differing age cohorts. These data may indicate that leadless pacing was considered attractive for younger patients with low pacing demand.

Shah *et al*.^6^ have reported on the experience of Micra in a paediatric population (≤21 years of age) with a mean age of 15 ± 4.1 years that included 63 patients with an implant success rate of 98%. This registry included 20 (32%) patients with congenital heart disease (CHD). Of the patients, 12.6% had the device implanted via the internal jugular approach with the remaining being by the more conventional femoral route. During a follow-up period of 9.5 ± 5.3 months, there were 10 (16%) complications that included a cardiac perforation, non-occlusive femoral venous thrombus, and one retrieval and replacement due to high pacing threshold. Wong *et al*.^7^ reported the implantation of the Aveir device in 10 patients (≤21 years) at a single centre with 90% being implanted using the jugular approach. The indication for pacing was intermittent complete heart block in 60% and sinus pauses in 40%. Predicted pacemaker longevity at follow-up was a median of 23.8 years. There were no complications in this patient cohort.

Strik *et al*.^8^ reported the experience of using Micra in 35 patients aged between 18 and 40 years in two French centres. The study reported a safety endpoint of 100% for freedom from system-related or procedure-related complications at 6 months and an efficacy endpoint of 94%. This endpoint was defined by a combination of a low (≤2 V) and stable (increase within 1.5 V) pacing capture threshold at 6 months. Of the patients, 37% had >40% pacing burden at 1 year.

Gulletta *et al*.^9^ reported Italian registry data of LP implantation with 6.2% of a cohort of 1154 patients being ≤50 years of age. The leading cause for implantation of an LP in the older population was for infective and vascular concerns. This contrasted with patient preference for an LP in the younger group. There were no differences in procedural/fluoroscopy times or complications between the groups. There was, however, a higher threshold in the younger patients both at discharge and follow-up (0.63 V vs. 0.5 V, *P* = 0.004).

The potential clinical value of using LP in a population under the age of 40 years has been recognized in an expert consensus statement in the UK on behalf of the British Heart Rhythm Society. Of LP implanters, 78% surveyed in this study recognized the value of considering LP implantation in those aged <40 years where there is a risk associated with TV pacing.^10^

## Potential advantages and disadvantages of leadless pacing in the young

### Long-term complications of vascular compromise with leads

In paediatric patients, transvenous leads can lead to long-term vascular complications, such as venous stenosis, thrombosis, and occlusion. These issues arise due to the mechanical stress imposed by the lead within small and often growing vasculature. Over time, the presence of the lead can result in endothelial injury and subsequent thrombus formation, significantly narrowing the vessel lumen. Paediatric patients are at a greater risk due to the smaller size of their veins and their growing anatomy, which exacerbates these complications. Several studies have documented the incidence of venous obstruction in patients with transvenous leads. Figa *et al*.^11^ reported a 18% incidence of partial or complete venous obstruction in children, indicating that lead-related vascular complications are not uncommon.

Bar-Cohen *et al*.^12^ highlighted that the incidence of venous occlusion in young children mirrors that seen in adults, and factors like patient age, body size, and lead characteristics at implantation do not clearly predict venous obstruction. The risk of vascular compromise increases with lead duration, and patient size, particularly in paediatric patients, where growth over time can cause additional strain on the vasculature.^13,14^

Silvetti *et al*.^15^ found that long-term complications in paediatric TP pacemaker recipients are lead-related, including lead fractures (6%), and are frequent in paediatric patients due to mechanical stress, a more active lifestyle, and a higher frequency of traumatic events, but thinner, more flexible leads can reduce the risk of vascular compromise. De Filippo *et al*.^16^ showed that using thinner leads in paediatric patients can help mitigate some of the risks of venous injury, though these designs still require long-term evaluation.

These findings suggest that venous injury at the time of implantation and lead diameter likely play critical roles in the development of vascular complications. Leadless pacemakers, which eliminate the need for transvenous leads, could potentially reduce these complications, offering a more viable long-term solution for paediatric patients.

### Complications and infections with generator changes

Infection rates among patients with cardiac implantable electronic devices vary between 1 and 19.9%, influenced by factors such as patient comorbidities, clinical profiles, procedural complexity, and the frequency or timing of re-interventions.^17^ Traditional pacemaker systems rely on a subcutaneous pulse generator, which requires periodic replacement due to battery depletion. Each replacement procedure carries inherent risks including infection at the generator pocket site. In paediatric patients, the risk is particularly concerning because they often require multiple generator changes over their lifetime. Infections related to generator changes can lead to serious complications, such as sepsis, endocarditis, and local abscesses, which in severe cases may necessitate complete system removal and even prolonged antibiotic therapy or additional surgeries. Moreover, children are at higher risk because their immune systems may respond differently, and the multiple invasive procedures throughout their life can cause lasting disruptions to the immune and vascular systems around the implant site. Cohen *et al*.^18^ reported a 7% infection rate in a cohort of 385 paediatric pacemaker patients, which included 19 cases (4.9%) of superficial infections, 9 cases (2.3%) of pocket infections, and 2 cases (0.5%) of isolated positive blood cultures. Pacemaker revisions were identified as an independent predictor of infection, with a relative risk of 2.5 (*P* < 0.01). Similarly, Klug *et al*.^19^ demonstrated that pacemaker infections were significantly more prevalent among young patients with CHD, with infection rates as high as 5.5% in this group compared with just 1.2% in older patients (under 40 years).

The advent of LP technology provides a promising alternative that could mitigate many of these risks. Leadless pacemakers, which are entirely self-contained within the heart and eliminate the need for subcutaneous pulse generators and transvenous leads, significantly reduce the risk of infections typically associated with generator pocket surgeries. Additionally, LPs are preloaded onto the delivery catheter, minimizing direct handling by the operator.^20,21^ With the growing development of rechargeable or extended-life battery technologies, future iterations of LPs may further extend the time between necessary interventions, thus offering a longer-lasting and less invasive solution for children requiring lifelong pacing.

### Tricuspid valve regurgitation

A major concern with traditional TP pacemakers is the risk of tricuspid valve regurgitation (TVR) worsening, caused by the pacemaker lead traversing the tricuspid valve and impairing its function.^22,23^ This can lead to progressive right-sided heart failure, particularly troubling for paediatric patients, who face long-term pacing and an increased risk of worsening valve dysfunction over time. Mazine *et al*.^24^ found a clear association between transvenous lead placement and significantly increased risk of persistent or recurrent TVR at late follow-up, highlighting the mechanical interference of the lead.

In a randomized trial, Garweg *et al*.^25^ compared the effects of the Micra LP with conventional single-chamber pacemakers. They observed that tricuspid and mitral valve function remained stable in the Micra group, while the conventional group experienced a significant worsening in valve function.

Although paediatric data are limited, avoiding transvenous leads in children could similarly reduce the risk of TVR. Early evidence points to LPs as a potentially safer option for younger patients requiring lifelong pacing, as they avoid the direct interaction with the tricuspid valve apparatus. Further studies are necessary to confirm these outcomes in paediatric populations.

### Disadvantages of leadless pacing in the young (life cycle issues)

Managing LP at the end of their life cycle presents unique challenges, particularly for younger patients who will likely need multiple replacements over their lifetime. Unlike traditional pacemakers, where generator replacements are relatively simple, LPs require either extraction or the addition of new devices. Extraction of LPs is technically feasible but can become increasingly difficult over time. The process often requires transvenous access, which may be complicated by fibrotic tissue, scarring, or anatomical changes, especially after long periods of implantation. Minami *et al*.^26^ indicated that extraction of LPs is possible with a retrieval success rate of 85%. However, it is a more prolonged and complex procedure compared with traditional transvenous leads. Moreover, repeated extraction in young patients may increase the risk of vascular or structural heart damage, further complicating future interventions. An alternative approach is to add new LPs without removing the old device. Leaving multiple devices inside the heart could lead to mechanical complications, especially in paediatric patients with smaller hearts. However, it is possible to implant up to three Micra devices in the same ventricle. This strategy allows for continued pacing without the need for extraction, but it is crucial to monitor the long-term impact of this approach, particularly regarding interactions with cardiac structures such as the tricuspid valve or right ventricular walls. Device longevity is also a key and crucial factor for young patients. Leadless pacemakers offer a battery life of 8–16 years, which may necessitate several replacements over a patient’s lifetime. Extending battery life or developing rechargeable options would significantly reduce the need for repeated interventions.^2,27^

### Lack of conventional TP pacing functionality

Traditional TP pacemaker systems offer continuous monitoring and detailed telemetry, which are essential for managing complex arrhythmias and evaluating device performance. Leadless pacemakers do not provide the same level of diagnostic capabilities as traditional pacemakers, such as detailed electrograms and AF monitoring. The absence of these diagnostic tools limits the ability to perform comprehensive cardiac assessments and monitoring of young patients with intricate arrhythmias.

### Evidence on femoral and jugular approaches

The femoral approach is the standard method for the implantation of LP. Most clinical studies have evaluated the safety and efficacy of this approach, showing high success rates and a relatively low complication profile. Studies have demonstrated a high success rate, with up to 99% successful implantation in early trials, and a low incidence of complications like vascular injury, thrombosis, and haematoma.^28–31^ Despite its success, the femoral approach is associated with specific risks, such as venous access complications (2.7%) and device dislodgement or migration, particularly during the learning curve of operators. Post-procedural recovery is generally quick, with patients often discharged within 24 h. Kiani *et al*.^32^ and Toon *et al*.^33^ showed and evaluated the feasibility and the safety of elective LP implantation practice implantation as a day-case procedure.

The jugular approach, although less commonly utilized, has been proposed as an alternative in certain anatomical conditions or when femoral access is not feasible. However, this technique is less supported by large-scale studies. Some case series, involving up to 100 adult patients, have suggested that the jugular route can be a viable option not only in patients with occluded femoral veins or significant vascular disease but also in unselected patients as an alternative to the femoral approach. These preliminary studies indicate that the jugular approach is safe and associated with a very low complication rate.^34–36^ Additionally, in paediatric patients, particularly those of smaller size or weight, the internal jugular approach for LP implantation has been explored. Several case reports and small series have documented successful pacemaker insertions via the jugular route in young patients.^37–41^ A case series described the implantation of LPs in eight paediatric patients weighing under 30 kg, where the internal jugular vein was used without complications, achieving up to 3 years of follow-up with positive outcomes.^37^ Another case involved a 28 kg child where the right internal jugular vein was selected due to concerns about femoral vein size, and the procedure was performed successfully with minimal complications.^38^ Additionally, in a 4-year-old patient weighing 16 kg, the jugular approach was also employed due to vessel size considerations and yielded a favourable post-operative outcome.^40^

### Requirement for imaging (pre/during/post-procedure)

Imaging plays a pivotal role in LP procedures, particularly in the paediatric population, where anatomical considerations and device positioning are critical. Pre-procedure imaging is essential for assessing the anatomical feasibility of LP implantation in children. Transthoracic echocardiography is typically employed to evaluate cardiac anatomy, venous structures, and right ventricular morphology, ensuring that the device can be positioned and secured appropriately. In addition, venous Doppler ultrasound or angiography is often used to evaluate vascular size and patency, particularly for the femoral or jugular veins. This is critical for ensuring that large delivery systems, such as the 27 F outer diameter required for LP, can be accommodated. Studies have shown that approximately 63% of paediatric cases document imaging (ultrasound or angiography) of the target vein prior to implantation to evaluate vessel suitability.^6^ While femoral veins in children are generally distensible and can tolerate large sheaths, patients with CHD or previous catheterizations may have stenosed or occluded veins, which necessitates pre-procedure imaging to determine optimal access routes. Fluoroscopy remains the standard imaging modality during the implantation of LP, providing real-time visualization of the device as it is advanced, positioned, and fixed within the heart. High-quality fluoroscopic guidance is crucial in the paediatric population to ensure optimal device orientation and minimize the risk of complications such as right ventricular wall perforation or device dislodgement. In a study with 108 patients, the application of right ventriculography during Micra™ LP implantation improved the accuracy of mid-septum positioning and reduced X-ray exposure compared with methods that did not use radiography.^42^ Post-implantation imaging is essential for confirming device stability, functionality, and monitoring potential complications such as pericardial effusion or dislodgement. Long-term follow-up imaging may include computed tomography or magnetic resonance imaging to evaluate the device’s impact on adjacent structures and overall cardiac function.

## Specific patient populations

The altered dynamic that LP brings to pacemaker therapy means that there are certain populations that require pacing that may be particularly suited to the leadless platform. This includes anatomical constraints that may make conventional TP pacing challenging or impossible, e.g. haemodialysis, vascular stenosis, or absence of access to the heart from a conventional superior approach. There are also particular patient populations where pacing may be required or considered desirable at a young age but challenged by the demands of many decades of pacing therapy that may be required. The cosmetic aspect of LP with the absence of surgical scars on the chest wall might be attractive to all ages including the young.

### Cardioinhibitory vasovagal syncope

Profound episodes of bradycardia or asystole associated with this condition can cause significant symptoms and have a major impact on individuals’ quality of life. However, the condition is frequently considered to be ‘benign’ in nature with very little evidence to support mortality associated with it. Consequently, pacing for vasovagal syncope (VVS) has remained a controversial therapy. Patients may present at all ages with the condition, and so, the decision-making process around whether to consider pacing as an option would be very different in an individual in their 20s or 30s compared with an individual in their 70s or 80s if considering TP pacing. Furthermore, it is recognized that for those presenting earlier in life, it is a condition that may improve or resolve with time.

A number of studies have examined the impact of TP pacing in patients with VVS and pauses with varying outcomes.^43–45^ Many of these small studies excluded patients under the age of 40 years, and so consequently, International Guidelines have excluded guidance on the use of pacemakers for the treatment of VVS in the under 40 population, though recognizing the rationale for this therapy in a younger population.^46^ In those studies that examined pacing in this population, dual-chamber pacing was studied. However, single-chamber leadless pacing might be considered to have particular benefits in this population. Use of a single-chamber device that would provide VVI pacing in the eventuality of profound bradycardia or asystole, logically, would have some haemodynamic benefit that may prevent syncope or mitigate symptoms to some extent. Furthermore, leadless pacing would preserve the vasculature in a young population. Pacing longevities in those where the device is used infrequently have been reported to be approaching 20 years with some devices. Leadless pacemakers have the unique ability to be programmed off and do not have to be explanted at the end of their battery life. This means that for some young individuals approaching the end of their LP device life, the device could be programmed off and the patient monitored to establish whether syncope associated with a cardioinhibitory response is still present.

A UK observational study presented 32 patients with VVS where LP was used as a treatment option. Patients were aged 37 ± 14 years (18–64 years) with symptom duration being 8 ± 8 years with an average annual frequency of syncope being 4 ± 6. This population had profound bradycardia/asystole with a mean documented pause of 13 ± 7 s. The follow-up was relatively short (404 ± 237 days), but 87% of patients were symptom free.^47^

Turagam *et al*.^48^ retrospectively studied 72 young patients (32 ± 5.5 years) that had frequent syncope (7.6 ± 3.4 events/year) and had been implanted with either an LP (*n* = 24) or dual chamber TP (*n* = 48). At 1 year, 22/24 patients in the LP group and 43/48 in the dual-chamber group were free from syncope (*P* = 0.7). There was no difference in adverse events between the two groups.

The limited evidence base for the use of LPs in the treatment of cardioinhibitory VVS in young patients is generally supportive but lacks large-scale prospective randomized studies. This therapy could be considered for patients under the age of 40 years but as part of a shared decision-making process.

### Adult congenital heart disease

Patients with adult CHD (ACHD) have a relatively high need for pacing therapy. This can be due to sinus node disease or high grade atrioventricular block as part of the natural history of some lesions or as a consequence of corrective/palliative surgery. The TP pacing can be challenging in this population for several reasons. Anatomy can be complex and, in some situations, can preclude conventional leads due to either obstructed/stenosed vessels or lack of superior access to the cardiac chambers as a consequence of surgery. Furthermore, the complications of TP leads may carry a higher degree of risk in this population when compared with a non-ACHD group, e.g. the risk of infection in an operated ACHD patient may have greater implications with either replaced valves or graft material that is more susceptible to infection. Historically, if TP pacing had been considered to be high risk, then epicardial systems have been deployed. These systems have been recognized to have higher incidences of elevated thresholds and fractured leads mandating repeated generator changes or additional leads. Leadless pacemaker in this population may offer an alternative option removing the requirement for leads and having the possibility to be deployed inferiorly, from the femoral veins, rather than superiorly using the subclavian approach.

Bassareo and Walsh reported a series of 15 patients with ACHD (37.5 ± 10.7 years) of varying complexity.^49^ Three patients (20%) had a systemic right ventricle, and two patients (13.3%) had single ventricle physiology. Two patients post-Mustard repair had the LP implanted in the left atrial appendage. Mean pacing threshold at 6 months was 0.60 V (range 0.38–1.13 V) with an R wave of 10.3 mV (range 3.25–19.4 mV). The median procedural duration was 62 min (range 59–64). One device had to be retrieved and repositioned after the threshold elevated post-tether cut. No other major device-related complications were reported.

The Fontan population of ACHD patients represents a particularly challenging patient group where the incidence of heart block requiring pacing can be as high as 40%. Commonly, direct access to the ventricle is not possible. Transvenous devices can have high rates of complications and so frequently epicardial devices are utilized. Goulden *et al*.^50^ present a series of four patients (aged 19–35 years) that were implanted with the Micra AV LP using different access routes including trans-fenestration and trans-carotid. Procedural time varied from 60 to 120 min, and all patients had clinically acceptable pacing parameters at 6-week follow-up.

Rahmat and Cortez^51^ described three patients (aged 27, 50, and 36 years) with ACHD that were implanted with the Aveir device. They had corrected Fallots, ventricular septal defect (VSD) repair, and interrupted aortic arch repair respectively. At follow-up, all pacing parameters were good and predicted longevity of the device was 22.7, >25, and 22.6 years.

### Neuromuscular diseases

Neuromuscular diseases are inherited and can affect the skeletal muscle but frequently the heart resulting in arrhythmias, cardiomyopathies, and conduction system disease in the young. Rapid progression to high degree AV block can be seen. Myotonic dystrophy types 1 and 2, Emery–Dreifuss, and limb girdle type 1B often present with conduction disease. European Society of Cardiology (ESC) guidelines give a IC recommendation for pacing in the instance of 2nd or 3rd degree block or an his-ventricle (HV) interval ≥ 70 ms. There is a IIb indication for pacing in myotonic dystrophy type 1 with a PR ≥ 240 ms or QRS duration ≥ 120 ms.^46^ Many of these patients will be young and without symptoms related to conduction system disease at this stage. Leadless pacemakers may represent an attractive option in this patient cohort ensuring safety from bradycardia/asystole but preserving the venous system without TP leads.

### Paediatric

Occasionally young children < 30 kg require pacing. TP pacing in this cohort has all the complications recognized in a conventional pacing population along with the added risks of compromise of the developing venous system and many prospective years of pacing therapy for the individual. There is some limited experience of the use of LP in this group of patients. Siddeek *et al*.^52^ reported a series of eight patients under 30 kg that received a Micra LP. The median age of this series was 7 years (range 2–10 years) with a median weight of 23.5 kg (10.9–29 kg). The internal diameter of the introducer sheath for the Micra device is 23 F. Consequently, the jugular vessels might be more attractive than femoral in this population. In this study, all patients were implanted using the internal jugular approach (five right, three left). The only major complication that was reported was a moderate pericardial effusion that was successfully drained. At follow-up, the median threshold was 0.5V@0.24 ms (range 0.38–1.63 V), median impedance of 605 Ω (range 475–680 Ω), and median R wave of 8.7 mV (range 5.6–13.5 mV). The Aveir device (38 mm) is longer than the Micra (25.9 mm) but in a single case report in a 23 kg 9-year-old was implanted successfully via the jugular approach without complication.^53^ Micra has also been reported in a neonate, being implanted on the epicardial surface of the heart using an adaptor to attach a bipolar epicardial lead in a neonate of 22 weeks of gestation, instead of a conventional generator that would be excessive in size.^54^ This was approved under ‘emergency use authorization’ by the US Food and Drug Administration.

## Conclusions

Transvenous pacing has been recognized to be an established therapy but associated with complications that can be associated with significant morbidity and mortality. Leadless pacing has been shown to reduce some complications. This makes leadless pacing particularly attractive in the younger age group that require pacing. The literature suggests that particular patient populations including patients with VVS, ACHD, and neuromuscular conditions associated with conduction system abnormalities and paediatric patients maybe effectively and safely treated with leadless devices. However, the literature is anecdotal with case series rather than with prospective randomized controlled studies that would inform the guidelines better.

## Data Availability

The data underlying this article will be shared on reasonable request to the corresponding author.
